# Wild-Type Phosphoribosylpyrophosphate Synthase (PRS) from *Mycobacterium tuberculosis*: A Bacterial Class II PRS?

**DOI:** 10.1371/journal.pone.0039245

**Published:** 2012-06-20

**Authors:** Ardala Breda, Leonardo K. B. Martinelli, Cristiano V. Bizarro, Leonardo A. Rosado, Caroline B. Borges, Diógenes S. Santos, Luiz A. Basso

**Affiliations:** 1 Instituto Nacional de Ciência e Tecnologia em Tuberculose (INCT-TB), Centro de Pesquisas em Biologia Molecular e Funcional (CPBMF), Programa de Pós-Graduação em Biologia Celular e Molecular, Pontifícia Universidade Católica do Rio Grande do Sul (PUCRS), Porto Alegre, Rio Grande do Sul, Brazil; 2 Programa de Pós-Graduação em Biologia Celular e Molecular, Pontifícia Universidade Católica do Rio Grande do Sul (PUCRS), Porto Alegre, Rio Grande do Sul, Brazil; University of Delhi, India

## Abstract

The 5-phospho-α-D-ribose 1-diphosphate (PRPP) metabolite plays essential roles in several biosynthetic pathways, including histidine, tryptophan, nucleotides, and, in mycobacteria, cell wall precursors. PRPP is synthesized from α-D-ribose 5-phosphate (R5P) and ATP by the *Mycobacterium tuberculosis prsA* gene product, phosphoribosylpyrophosphate synthase (*Mt*PRS). Here, we report amplification, cloning, expression and purification of wild-type *Mt*PRS. Glutaraldehyde cross-linking results suggest that *Mt*PRS predominates as a hexamer, presenting varied oligomeric states due to distinct ligand binding. *Mt*PRS activity measurements were carried out by a novel coupled continuous spectrophotometric assay. *Mt*PRS enzyme activity could be detected in the absence of P_i_. ADP, GDP and UMP inhibit *Mt*PRS activity. Steady-state kinetics results indicate that *Mt*PRS has broad substrate specificity, being able to accept ATP, GTP, CTP, and UTP as diphosphoryl group donors. Fluorescence spectroscopy data suggest that the enzyme mechanism for purine diphosphoryl donors follows a random order of substrate addition, and for pyrimidine diphosphoryl donors follows an ordered mechanism of substrate addition in which R5P binds first to free enzyme. An ordered mechanism for product dissociation is followed by *Mt*PRS, in which PRPP is the first product to be released followed by the nucleoside monophosphate products to yield free enzyme for the next round of catalysis. The broad specificity for diphosphoryl group donors and detection of enzyme activity in the absence of P_i_ would suggest that *Mt*PRS belongs to Class II PRS proteins. On the other hand, the hexameric quaternary structure and allosteric ADP inhibition would place *Mt*PRS in Class I PRSs. Further data are needed to classify *Mt*PRS as belonging to a particular family of PRS proteins. The data here presented should help augment our understanding of *Mt*PRS mode of action. Current efforts are toward experimental structure determination of *Mt*PRS to provide a solid foundation for the rational design of specific inhibitors of this enzyme.

## Introduction

Tuberculosis (TB) is a chronic infectious disease caused mainly by *Mycobacterium tuberculosis*, being the second leading cause of mortality by infectious diseases in human populations, killing about 1.7 million people worldwide in 2009 [1]. One third of the world population is estimated to be infected with latent TB. The latter is worsened by the spread of HIV-TB co-infection, which can lead to increased rates of TB reactivation, being up to 30% of deaths among HIV positive subjects caused by the TB bacilli [2]. TB infection is treated by a combination of four drugs that act upon different molecular targets [3]. The treatment regimen includes six month therapy with rifampicin and isoniazid, supplemented with pyrazinamide and ethambutol in the first two months [1]. In recent years, *M. tuberculosis* isolates resistant to one or more of these drugs have been spreading, which seriously hampers the success of measures to control TB [4]. The increasing incidence of TB has been paralleled by a rapid increase of cases caused by multi-drug resistant (MDR-TB) and extensively-drug resistant *M. tuberculosis* strains (XDR-TB), with estimated cases and annual deaths worldwide of, respectively, of 0.5 million and 100,000 for MDR-TB, and 35,000 and 20,000 for XDR-TB [5], [6]. Recently, TB infection with totally resistant strains (TDR-TB), which are resistant to all first and second line classes of anti-TB drugs tested, have been isolated in Iran and India [7], [8]. There is an urgent need to develop new therapeutic strategies to combat TB. Strategies based on the selection of new targets for antimycobacterial agent development include elucidation of the role played by proteins from biochemical pathways that are essential for mycobacterial growth [9].

**Figure 1:**
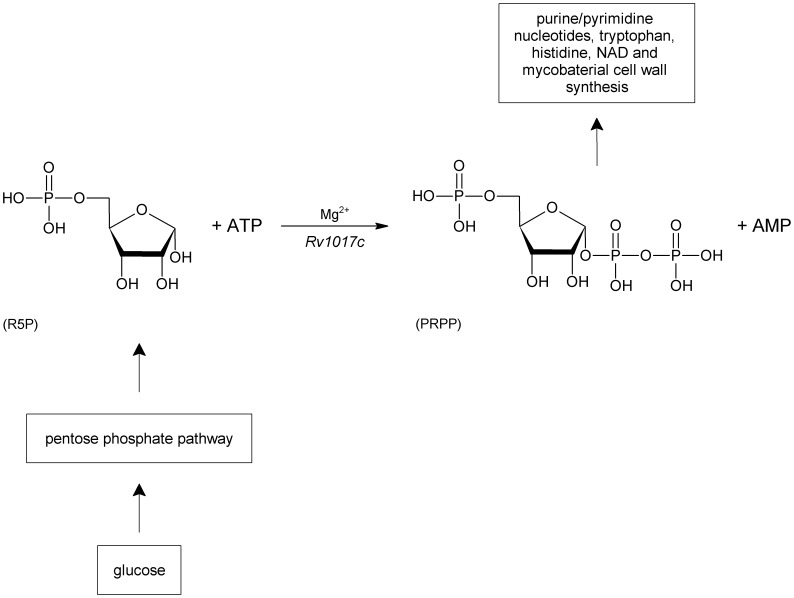
Chemical reaction catalyzed by *Mt*PRS (Rv1017c). This figure also shows the metabolic source of R5P and the biosynthetic pathways in which the reaction product PRPP plays central roles.

Phosphoribosylpyrophosphate synthase (PRS; EC 2.7.6.1) plays central roles in a number of cellular processes, catalyzing the synthesis of 5-phospho-α-D-ribose 1-diphosphate (PRPP; α-D-5-phosphoribosylpyrophosphate; α-D-ribosyl diphosphate 5-phosphate). PRS enzymes catalyze, in the presence of Mg^2+^, the transfer of β,γ-diphosphoryl moiety of adenosine 5′-triphosphate (ATP) to C1-hydroxyl group of α-D-ribose 5-phosphate (R5P), yielding PRPP [10], [11] (**Figure 1**). PRPP is an essential metabolite for a number of distinct biochemical pathways including *de novo* and salvage pathways of purine and pyrimidine nucleotide synthesis, and biosynthesis of NAD, histidine and tryptophan [12]–[14]. PRPP is also associated with cell integrity in *Saccharomyces cerevisiae* [15]. The yeast genome encodes five distinct *prs* genes, whose products are combined to form hetero-oligomeric catalytic active PRS, with possible role in plasma membrane stability [16]. In Corynebacteriaceae, such as mycobacteria, PRPP is a co-substrate for the synthesis of polyprenylphosphate-pentoses, which are the source of arabinosyl residues of arabinogalactan, component of the mycobacterial cell wall, and lipoarabinomannan, a highly immunogenic lipoglycan that is involved in modulating the host immune response [17], [18].

PRS enzymes usually require Mg^2+^•ATP as diphosphoryl group donor. The PRS proteins from *Escherichia coli* [19], *Salmonella typhimurium* [20] and mammals [21] have been shown to also require a second free Mg^2+^ ion for increased catalytic rates. PRS enzymes from these organisms, as well as from *Bacillus subtilis* [22], are representative of Class I (also known as “Classical=") PRS proteins, with hexameric quaternary structure, allosteric inhibition by purines ribonucleoside diphosphate (adenosine 5′-diphosphate, ADP; and guanosine 5′-diphosphate, GDP), specificity for ATP (or dATP) as diphosphoryl group donor, and requirement of inorganic phosphate (P_i_) for enzyme activity [23]. The three-dimensional structures of PRS enzymes from *B. subtilis* (PDB ID: 1IBS) [21] and *Homo sapiens* (PDB ID: 2H06) [11] demonstrate that the functional enzyme is a hexamer of identical subunits, associated two by two, where each monomer is composed by two domains, both with high topological similarity to the type I family of phosphoribosyltransferases [24]. In addition, there is conservation of amino acid residues in the PRPP substrate binding site [22]. Class II PRS proteins share several structural characteristics with Class I enzymes. However, Class II PRSs are characterized by not being dependent on P_i_ for activity, have broad specificity for diphosphate donors (including guanosine 5′-triphosphate, GTP; cytosine 5′-triphosphate, CTP; and uridine 5′-triphosphate, UTP), and are not allosteric inhibited by purines ribonucleosides diphosphate [23], [25]. Class II PRS proteins appear to be specific for plants as they have been identified in spinach [26] and *Arabidopsis thaliana* isozymes 3 and 4 [27]. Nevertheless the PRS enzyme from pathogenic Gram negative enterobacteria *S. typhimurium* was reported as using GTP, ITP, CTP and UTP in addition to ATP as substrate [28]. More recently, a PRS enzyme from the archeon *Methanocaldococcus jannaschii* has been shown to be tetrameric (PDB ID: 1U9Y), activated by P_i_, non-allosteric inhibited by ADP, and that employs ATP as diphosphate donor [25]. These findings prompted the proposal that *M. jannaschii* PRS belongs to a new Class III of PRPP synthases [25].

Here we describe cloning of *prsA* (Rv1017c) from *M. tuberculosis*; and expression, purification, molecular and kinetic characterization of the non-tagged recombinant PRS (*Mt*PRS). Glutaraldehyde cross-linking results indicate that the oligomeric state of *Mt*PRS is predominantly hexameric in solution. However, the presence of ligands appears to stabilize alternative oligomeric states. *Mt*PRS activity was assessed by a novel coupled continuous spectrophotometric assay that measures the decrease in orotate concentration catalyzed by *M. tuberculosis* orotate phosphoribosyltransferase (*Mt*OPRT) due to PRPP formation by recombinant *Mt*PRS enzyme activity. Steady-state data indicate that *Mt*PRS can use both pyrimidine and purine nucleosides triphosphate as diphosphoryl group donors (broad specificity). In addition, enzyme activity measurements show that *Mt*PRS is catalytically competent in the absence of P_i_. These data suggest that *Mt*PRS belongs to Class II PRS family, as plant homologues, even though the primary amino acid structure is indicative of structural resemblance to Class I PRS. Equilibrium binding data are also presented suggesting that *Mt*PRS mechanism is likely random order of substrate addition for purine diphosphoryl donors and ordered addition of pyrimidine diphosphoryl donors, with ordered release of products in which PRPP dissociation is followed by the purine or pyrimidine nucleoside monophosphate products. The *prsA*-encoded protein has been predicted to be essential for *in vitro* growth of *M. tuberculosis* based on transposon-site hybridization studies [29]. More recently, PRS from *Corynebacterium glutamicum*, a model organism used to study *M. tuberculosis* cell physiology, has been shown to be essential for the maintenance of cellular integrity [30]. The results presented here are discussed in light of previous reports on *Mt*PRS [30], [31], and should thus contribute to a better understanding of *Mt*PRS mode of action.

## Methods

### Gene Amplification

The *prsA* gene (Rv1017c) was PCR amplified from total genomic DNA of *M. tuberculosis* H37Rv strain using specific primers designed to contain *Nde*I (primer sense 5′GCCATATGAGCCACGACTGGACCGATAATCG3′) and *Bam*HI (primer antisense 5′GCGGATCCTCATGCGTCCCCGTCGAAAAGT3′) restriction sites (underlined). An internal restriction site for *Nde*I was removed from the gene sequence by site-directed mutagenesis at codon position 170, in which a thymine was replaced with a cytosine at codon’s third position (CAT to CAC), resulting in a sense mutation that maintained a histidine amino acid at this position. Dimethyl sulfoxide (DMSO) was added to the PCR reaction at final concentration of 10%. Amplified *prsA* gene was cloned into pET-23a(+) expression vector (Novagen). The integrity of constructs was confirmed in all cases by appropriate selections and digests with restriction enzymes (New England Biolabs). Inserted sequences were confirmed by automated DNA sequencing.

### Expression and Purification of Recombinant *Mt*PRS

**Figure 2:**
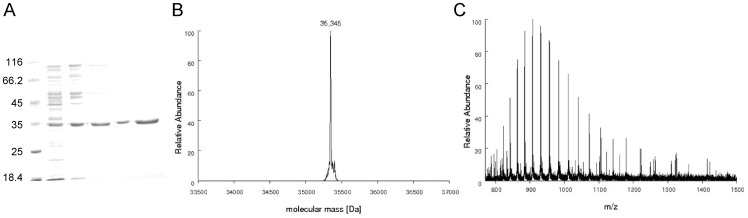
A) MtPRS purification steps. Lane 1: Protein Marker – Fermentas; lane 2: crude extract; lane 3: sample loaded on Q-Sepharose; lane 4: sample eluted from Q-Sepharose; lane 5: sample eluted from Superdex 200; lane 6: protein fraction eluted from Mono Q HR anion exchange step showing homogeneous recombinant *Mt*PRS (approximately 35 kDa). **B**) Determination of *Mt*PRS molecular mass by mass spectrometry analysis. Deconvoluted spectra of *Mt*PRS resulted in a peak corresponding to the molecular mass of 35,345 Da. **C**) ESI-FTMS spectra showing the charge distribution obtained for *Mt*PRS, spanning from charge state 25+ to 45+.

Competent *E. coli* BL21(DE3) (Novagen) cells were electroporated with pET-23a(+)::*prsA* recombinant vector and selected on Luria-Bertani (LB) agar plates containing 50 µg mL^−1^ ampicillin. Aliquots of a 5 mL cell culture grown from a single colony were used to inoculate 500 mL of Terrific Broth (TB) medium supplemented with 50 µg mL^−1^ ampicillin, grown at 37°C and 180 rpm to an optical density (OD_600 nm_) of 0.4–0.6. At this growth stage, culture temperature was lowered to 30°C and protein expression was carried out without isopropyl-β-D-thiogalactopyranoside (IPTG) induction, for 24 hours. Cells were harvested by centrifugation (11,800 *g*) for 30 min at 4°C and stored at −20°C. Protein purification was performed by Fast-Performance Liquid Chromatography (FPLC) on Äkta Purifier System (GE HealthCare) at 4°C. Cell pellet (4 g) was suspended in 40 mL of buffer A (Tris HCl 50 mM pH 7.5) and stirred for 30 min. Cells were disrupted by sonication (12 pulses of 10 sec, with intervals of 1 min off) in presence of 0.2 mg mL^−1^ lysozyme (Sigma Aldrich), and the clarified supernatant (48,000 *g* for 30 min in all cases) was further treated with 1% (wt/vol) streptomycin sulfate (Sigma-Aldrich). The latter supernatant was treated with 2.5 M ammonium sulfate and the resulting precipitate was suspended in 40 mL of buffer A (crude extract). The crude extract was loaded on a Q-Sepharose Fast Flow anion exchange column (GE Healthcare) equilibrated with buffer A. Adsorbed material was eluted with 0% to 50% linear gradient of Tris HCl 50 mM NaCl 1 M pH 7.5 (buffer B) at 1 mL min^−1^ flow rate. Fractions containing *Mt*PRS, as inferred by 12% SDS-PAGE polyacrilamide gel electrophoresis stained with Coomassie Brilliant Blue [32], were pooled, concentrated and loaded on a Superdex 200 size exclusion column (GE Healthcare) previously equilibrated with buffer A. Proteins were eluted in isocratic conditions and fractions containing *Mt*PRS were loaded on a Mono Q HR 16/10 anion exchange column (GE Healthcare) equilibrated with buffer A. Proteins were eluted with 0% to 100% linear gradient of buffer B, at 1 mL min^−1^ flow rate (**Table 1**). Homogeneous *Mt*PRS eluted at approximately 430 mM NaCl (**Figure 2A**). Fractions containing homogeneous *Mt*PRS were pooled, dialyzed against buffer A, concentrated, and stored at −80°C up to 7 months without any loss of enzyme activity.

**Table 1:** Purification of *Mt*PRS from 4 g of wet cell paste of *E. coli* BL21(DE3) host cells.

Purification step	Total protein (mg)	Specific activity(U mg^−1^)	Total enzyme activity (U)	Yield %	Purification fold
crude extract	524	0.028	145	100	1
Q-Sepharose FF	19.6	0.518	10.16	7	18.5
Superdex 200	19	0.220	4.17	2.8	7.9
MonoQ 16/10	3.5	1.16	4.06	2.8	41.4

### *Mt*PRS Identification by Mass Spectrometry

LC-MS/MS peptide mapping experiments were performed to confirm the identity of *Mt*PRS samples. Briefly, the purified samples were digested with trypsin using a protocol adapted from [33], and the digested peptides were chromatographically separated (Kinetex 2.6 µm C18 core-shell particles - Phenomenex, Inc.) using a nanoLC Ultra system (nanoLC Ultra 1D plus, Eksigent, USA) connected to a LTQ-Orbitrap hybrid mass spectrometer (Thermo Electron Corporation, San Jose, CA). The chromatographic method used a flow rate of 300 nL min^−1^ with a step gradient from mobile phase A containing 0.1% formic acid in water to mobile phase B containing 0.1% formic acid in acetonitrile (0–2% B over 5 min; 2–10% B over 3 min; 10–60% B over 60 min; 60–80% B over 2 min; 80% B isocratic for 10 min; 80–2% B over 2 min; and 2% B isocratic for 8 min). MS/MS fragmentation was performed using collision-induced dissociation (CID) with an activation Q of 0.250, an activation time of 30.0 ms, 35% of normalized collision energy, and an isolation width of 1.0 Da. LC-MS/MS data were compared with theoretical MS/MS spectra obtained from in-silico tryptic digests of the *M. tuberculosis* H37Rv proteome (ftp://ftp.ncbi.nih.gov/genomes).

### Determination of *Mt*PRS Subunit Molecular Mass

Purified *Mt*PRS samples were desalted, reconstituted in methanol 50%/formic acid 1% and directly injected into an IonMax electrospray ion source. The electrospray source parameters were as follows: positive ion mode, 5 kV of applied voltage to the electrospray source, 37.6 V of capillary voltage, 310°C of capillary temperature, and 109 V of tube lens voltage. Full spectra (770–2000 m/z range) were collected during 20 min on a Thermo Orbitrap Discovery XL in profile mode at a nominal resolution r  = 30,000 at m/z 400 using FT automatic gain control target value of 1,000,000 charges. The average spectrum was processed with the software MagTran [34] for charge state deconvolution.

### *Mt*PRS Quaternary Structure Assessment by Cross-linking Studies

Cross-linking studies of the protein’s oligomeric states were performed as described by Fadouloglou *et al*. [35], using crystallization supports with 120 µL of 25% (v/v) glutaraldehyde acidified with HCl in the reservoir. A cover slip was used to seal the reservoir, containing a 10 µL drop of protein suspension (0.3 mg mL^−1^ homogeneous recombinant *Mt*PRS in buffer A) in its apo form and incubated with P_i_ 50 mM, both in presence and absence of ATP 5 mM, R5P 5 mM, and ADP 5 mM. The plates were incubated at 30°C for different time intervals and protein drops were subsequently analyzed by 12% SDS-PAGE.

### *Mt*PRS Activity Assays

All chemicals were purchased from Sigma Aldrich. All enzyme activity assays were performed in triplicate. *Mt*PRS activity was measured by a coupled continuous spectrophotometric assay in quartz cuvettes using a UV-visible Shimadzu spectrophotometer UV2550 equipped with a temperature-controlled cuvette holder. *Mt*PRS reaction (ATP + R5P → PRPP + AMP) was coupled to *M. tuberculosis* orotate phosphoribosyltransferase (*Mt*OPRT, EC 2.4.2.10) forward reaction (OA + PRPP → OMP + PP_i_), in which PRPP synthesis can be monitored by the decrease in orotate (OA) concentration at 295 nm, for 60 sec at 25°C, using an extinction coefficient value of 3950 M^−1^cm^−1^ [36], when ATP was the diphosphoril group donor of the reaction catalyzed by *Mt*PRS. When GTP, CTP and UTP were used as substrates for *Mt*PRS enzyme activity measurements, the decrease in OA concentration was monitored at 303 nm, for 60 sec at 25°C, using an extinction coefficient of 2200 M^−1^cm^−1^ [37] due to strong absorption of these nucleosides triphosphate at 295 nm. Homogeneous recombinant *Mt*OPRT was obtained as described elsewhere [38]. Coupled assay conditions so as the indicator enzyme (*Mt*OPRT) did not limit the primary reaction (*Mt*PRS) were employed according to [39] and [40]. The reaction mixture (500 µL) contained 1.8 U *Mt*OPRT, 300 µM OA, 20 mM MgCl_2_, in Tris HCl 50 mM pH 8.0. Reaction was started by addition of *Mt*PRS (0.24–1.2 µM). One unit of *Mt*PRS is defined as the amount of enzyme necessary to convert 1 µmol of R5P to PRPP per min. Effect of P_i_ over *Mt*PRS activity was assessed by varying P_i_ concentration (10–50 mM) in the reaction conditions described above.

Apparent steady-state kinetic constants, *K_M_^app^* and *V_max_^app^*, were determined by fitting the data for each substrate pairs to Henri-Michaelis-Menten equation, **Eq. (1)** [41], in which *v*, *V_max_*, [*S*], and *K_M_* represent, respectively, steady-state reaction rate, maximum reaction rate, substrate concentration, and Henri-Michaelis-Menten constant for substrate S. The *k_cat_* values and substrate inhibition constant (*K_i_*) were calculated, respectively, from **Eq. (2)** [42] and **Eq. (3)** [41]; in which *k_cat_* and [*E*]*_t_* correspond to, respectively, catalytic constant, or turnover number, and total enzyme concentration, for **Eq. (2)**. For **Eq. (3)**, *K_i_* represents the dissociation constant for the inhibitory complex, and the remaining variables are as for **Eq. (1)**. Data analysis was performed using SigmaPlot 10 Software.
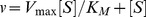
(1)

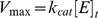
(2)

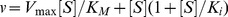
(3)


### Inhibition Assays

Inhibition assays were performed at fixed-saturating concentrations of R5P (60 µM) and ATP (300 µM), in either absence or presence of varied concentrations of ADP (20 µM to 1.5 mM), GDP (500 µM to 5 mM) or UMP (1 mM to 12 mM). Reaction was started by addition of 0.24 µM *Mt*PRS, under assay conditions described above for substrate pair ATP/R5P. All measurements were performed in triplicate. The concentration of inhibitor required to reduce the fractional enzyme activity to half of its initial value in the absence of inhibitor (IC_50_) was obtained from fitting the data to **Eq. (4)** for partial inhibition [41], in which *y* is the fractional activity of the enzyme in the presence of inhibitor at concentration [I]; *y*_(max)_ is the maximum value of *y* observed at [I]  = 0; and *y*_min_ is the minimum limiting value of *y* at high inhibitor concentrations.
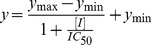
(4)


### Intrinsic Tryptophan Fluorescence (ITF) Spectroscopy

Intrinsic tryptophan fluorescence titration was carried out to assess binary complex formation at equilibrium between *Mt*PRS and either substrate(s) or product(s) at 25°C [43]. All substrates (R5P, ATP, GTP, UTP and CTP), products (AMP and PRPP) and the enzyme were dissolved in buffer A containing MgCl_2_ 20 mM. Fluorescence titrations were performed by making microliter additions of substrates and products at varying stock concentrations to 1 mL of *Mt*PRS 3 µM, with a maximum dilution of 6%. Ligand concentration ranges were as follow: R5P 0.99–126.83 µM; ATP 0.9–169.65 µM; GTP 0.9–309.24 µM; UTP 0.9–389.25 µM; CTP 0.9–389.25 µM; AMP 0.99–389.25 µM; and PRPP 0.99–389.25 µM. After each ligand titration, the mixture was stirred for 3 minutes to ensure equilibrium binding prior to ITF measurements. Measurements of ITF of *Mt*PRS employed excitation wavelength values of 292 nm (R5P) and 295 nm (PRPP, AMP, ATP, GTP, UTP and CTP), and the emission wavelength ranged from 300 nm to 400 nm (maximum *Mt*PRS λ_EM_ = 336 nm). In the binding experiments, different slits for, respectively, the excitation and emission wavelengths were employed: 1.5 nm and 5 nm for R5P, 1.5 nm and 10 nm for binding of ATP, GTP, UTP and CTP, and 1.5 nm and 10 nm for the products AMP and PRPP. Control experiments were performed in the same conditions in the absence of *Mt*PRS to verify any inner filter effect, and the values found in the control experiments were subtracted from those obtained in the presence of the enzyme. No corrections for effects of protein dilution on ITF upon addition of buffer A containing MgCl_2_ 20 mM to *Mt*PRS were necessary. Data from equilibrium fluorescence spectroscopy were fitted to **Eq. (5)** for hyperbolic binding isotherms, in which *F* is the observed fluorescence signal; *F*_max_ is the maximal fluorescence intensity; and *K_D_* represents the dissociation constant for binding of substrate and/or product to *Mt*PRS. Sigmoidal binding data were fitted **Eq. (6)** [44], in which *F* is the observed fluorescence signal, *F*_max_ is the maximal fluorescence intensity, *n* is the Hill coefficient, and *K’* is a constant comprising interaction factors and the intrinsic dissociation constant [42].
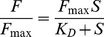
(5)

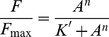
(6)


## Results

### Cloning, Expression and Purification of Recombinant *Mt*PRS

Automated DNA sequencing confirmed the identity and integrity of the pET-23a(+)::*prsA* construct. Recombinant *Mt*PRS protein was purified to homogeneity (**Figure 2A**) by a three-step chromatographic protocol, with 2.8% yield and approximately 41 fold purification (**Table 1**). Desorption of recombinant *Mt*PRS from Q-Sepharose Fast Flow anion exchange column occurred at approximately 390 mM salt concentration, with removal of substantial amount of contaminants from the total protein sample. Salt removal after size exclusion step led to an activity loss that was reverted after homogeneous *Mt*PRS elution from Mono Q HR at 430 mM salt concentration. Identity of recombinant *Mt*PRS was assigned by LC-MS/MS peptide mapping experiments, with coverage of 61% of its primary sequence.

### Mass Spectrometry Analyses

#### LC-MS/MS peptide mapping experiments

Apparently homogeneous *Mt*PRS samples were desalted, digested with trypsin, and the peptide mixtures subjected to LC-MS/MS analysis as described in the Methods section. 188 spectra were obtained and identified with 27 different peptides derived from the trypsin digestion of the *Mt*PRS protein. These peptides covered 61% of the *Mt*PRS sequence.

#### Molecular mass determination by mass spectrometry

The spectra of intact *Mt*PRS samples were recorded with the Orbitrap analyzer for molecular mass determination as described in the Methods section. Peaks corresponding to different charge states spanning from 25+ to the multiple charge state 45+ were detected. From the deconvoluted spectra, a value of 35,345 Da was determined for the average molecular mass of *Mt*PRS, consistent with the post-translational removal of the N-terminal methionine (theoretical subunit molecular mass of 35,477.47 Da with methionine and 35,346.28 without methionine) (**Figure 2B**). As the value for subunit molecular mass of *E. coli* PRS is 34,218.2, the mass spectrometry analysis also demonstrates that the homogeneous protein is indeed recombinant *Mt*PRS.

### *Mt*PRS Quaternary Structure Assignment

**Figure 3:**
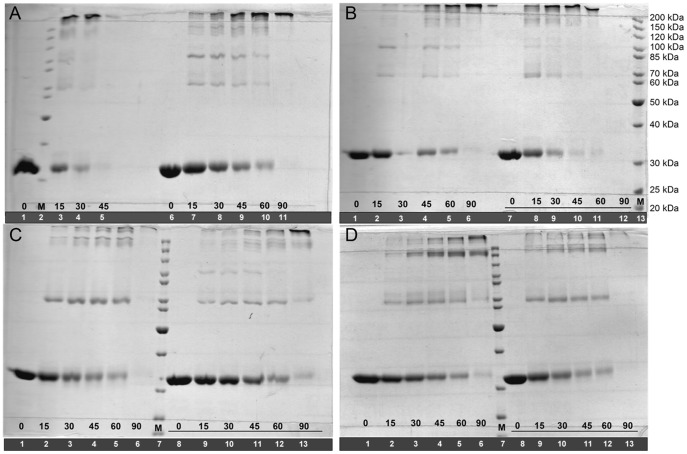
*Mt*PRS quaternary structure assignment by glutaraldehyde cross-linking experiments. Incubation times (numbers in black) are shown at the bottom of each lane. Underlined incubation times indicate the presence of 50 mM P_i_ in the reaction mixtures. Lane numbers are in white in a solid black background. M: Page Ruler Marker (Fermentas). **A)** Apo *Mt*PRS. **B)** *Mt*PRS incubated with R5P 5 mM. **C)** *Mt*PRS incubated with ATP 5 mM. **D)** *Mt*PRS incubated with ADP 5 mM.

*Mt*PRS quaternary structure could not be assigned by analytical HPLC gel filtration chromatography due to formation of protein aggregates under the experimental conditions described elsewhere [45]. Cross-linking experiments of apo *Mt*PRS were thus pursued and indicates that there is a shift from monomeric, intermediate multi oligomeric states, to predominantly hexameric forms (approximately 220 kDa) after 45 min incubation time in the absence of P_i_ (**Figure 3A**, lanes 1, 3–5). Intermediate oligomers, mostly dimers (∼70 kDa), trimers (∼100 kDa) and tetramers (∼150 kDa) could also be visualized on Coomassie Brilliant Blue stained gels. Although the presence of P_i_ 50 mM did not change this oligomerization profile, it appears to have delayed the shift of *Mt*PRS to hexameric forms (**Figure 3A**, lanes 6–11). Pre-incubation with R5P 5 mM in either absence or presence of P_i_ 50 mM appears to have no noticeable effects on shifting the oligomeric states of *Mt*PRS (**Figure 3B**) as the profiles are similar to the apo form of *Mt*PRS (**Figure 3A**). Pre-incubation with ATP 5 mM seems to stabilize *Mt*PRS dimeric state (**Figure 3C**), whereas pre-incubation with ADP 5 mM suggests that there is an increase in the tetrameric state of *Mt*PRS over time (**Figure 3D**), both in the absence and presence of P_i_ 50 mM.

### Enzyme Activity, Substrate Specificity and Inhibition Assays

**Figure 4:**
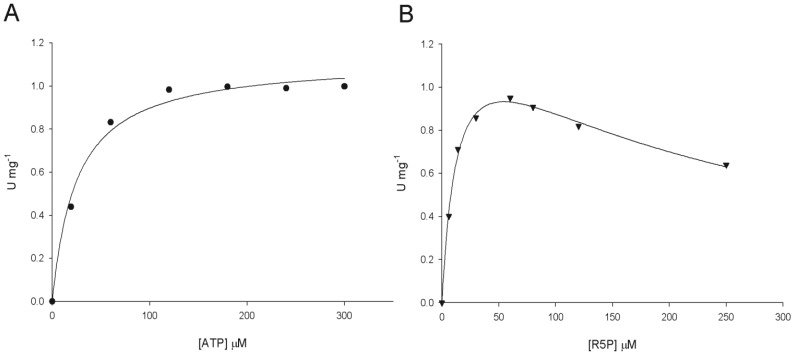
Apparent steady-state kinetic constants for *Mt*PRS, measured under standard assay conditions (Methods), for substrate pair ATP/R5P. A) Varied ATP concentrations in presence of 60 µM R5P. B) Varied R5P concentrations in presence of saturating ATP (300 µM).

*Mt*PRS enzyme activity could be detected in the absence of P_i_, and in the presence of varying concentrations of ATP diphosphoryl group donor at fixed 60 µM of R5P (**Figure 4A**). When substrate ATP was fixed at saturating concentration (300 µM) and enzyme activity measurements at varying R5P concentrations were carried out, substrate inhibition was observed at R5P concentration values larger than 60 µM (**Figure 4B**). Addition of 10–50 mM of P_i_ to the assay mixtures abrogated *Mt*PRS enzyme activity detection due to inhibition of coupled enzyme *Mt*OPRT (*data not shown*), likely due to chelating effect of PO_4_^3−^ anions on Mg^2+^ cations [46]. As Mg^2+^•PRPP is the true substrate of *Mt*OPRT [38], addition of P_i_ into the reaction mixture would result in no formation of the true substrate and ensuing lack of activity of *Mt*OPRT coupled enzyme. Accordingly, all *Mt*PRS enzyme activity assays henceforth described were carried out in the absence of P_i_.

**Figure 5:**
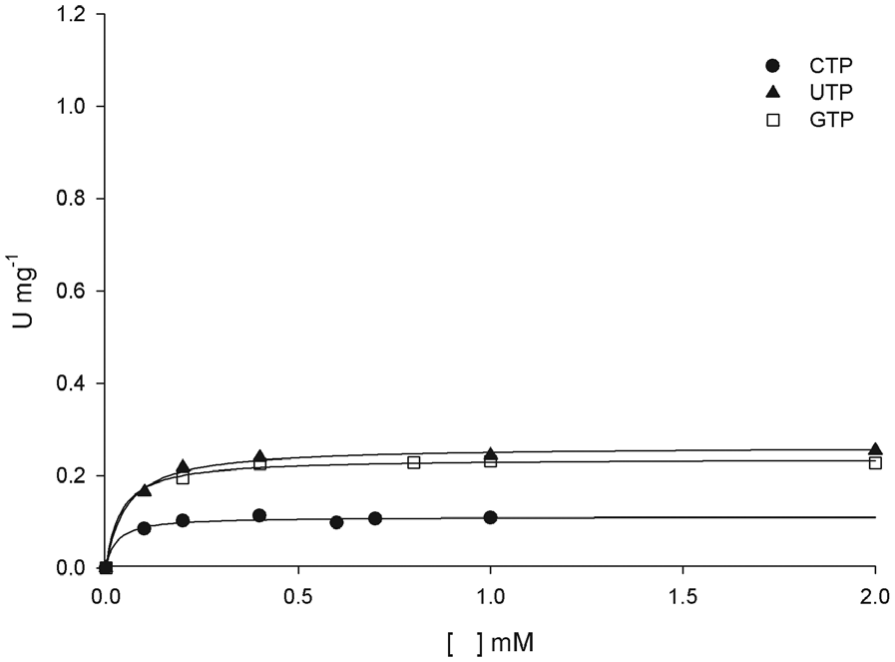
Apparent steady-state kinetic constants for *Mt*PRS, measured under standard assay conditions (Methods), for substrates GTP, CTP and UTP, varied in the presence of fixed-saturating concentration of R5P (60 µM).

*Mt*PRS enzyme activity could be detected when, under the same experimental conditions, the ATP diphosphoryl group donor was replaced with either purine (GTP) or pyrimidine (CTP and UTP) nucleoside 5′-triphosphates (**Figure 5**). Although the values for the catalytic rate constants (*k*_cat_) of GTP, UTP and CTP are lower than the value for ATP, the apparent overall dissociation constant (*K_M_*) values are somewhat similar (**Table 2**). The lower *k*_cat_ values and similar *K_M_* values result in lower values for the specificity constant (*k*_cat_/*K_M_*) of GTP, UTP and CTP in comparison to ATP (**Table 2**). These results indicate that *Mt*PRS has broad substrate specificity being able to use ATP, GTP, CTP and UTP as diphosphoryl group donors.

**Table 2:** Apparent kinetic parameters for *Mt*PRS reaction.

Substrate pair	Kinetic parameters				
	*K_M_* (µM)	*V_max_* (µmol^−1^ min^−1^ mg^−1^)	*k_cat_* (s^−1^)	*k_cat_/K_M_* (M^−1^ s^−1^)	*K_i_* (µM)
**ATP/R5P**	25 (±4)	1.12 (±0.03)	0.66 (±0.02)	26 (±4)×10^3^	–
**R5P/ATP**	14 (±2)	1.41 (±0.07)	0.83 (±0.04)	59 (±8)×10^3^	211 (±28)
**GTP/R5P** *	37 (±9)	0.237 (±0.004)	0.140 (±0.002)	3.8 (±0.9)×10^3^	–
**UTP/R5P** *	52 (±7)	0.264 (±0.005)	0.155 (±0.003)	3.0 (±0.4)×10^3^	–
**CTP/R5P** *	26 (±1)	0.111 (±0.003)	0.065 (±0.001)	2.5 (±0.1)×10^3^	–

* Fixed R5P concentration at 60 µM.

**Figure 6:**
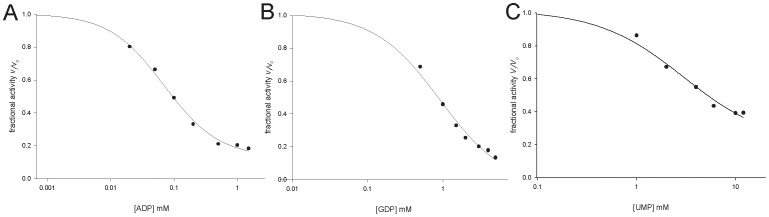
Inhibition of *Mt*PRS enzyme activity by A) ADP; B) GDP; and C) UMP. *Mt*PRS expressed as its fractional activity; and ADP, GDP and UMP concentrations were plotted on log scale.

Addition of both ADP (**Figure 6A**) and GDP (**Figure 6B**) to *Mt*PRS reaction mixture (ATP and R5P fixed at, respectively, 300 µM and 60 µM, under assay conditions described in the Methods section) resulted in partial inhibition of enzyme activity. The data on partial enzyme inhibition were fitted to **Eq. (4)**, yielding IC_50_ values of, respectively, 0.07 (±0.01) mM and 0.9 (±0.1) mM for ADP and GDP. Addition UMP to the reaction mixture also resulted in partial inhibition of *Mt*PRS, and data fitting to **Eq. (4)** yielded an IC_50_ value of 3.0 (±0.8) mM (**Figure 6C**).

To ascertain whether or not these experimental data were due to effects on *Mt*PRS activity and not on *Mt*OPRT coupled enzyme, measurements of activity of the latter enzyme were performed in the presence of the diphosphoryl group donors (ATP, GTP, CTP, and UTP), and nucleoside diphosphate or monophosphate inhibitors (ADP, GDP and UMP). The presence of these compounds in the assay mixtures employed in the coupled assays did not have any effect on *Mt*OPRT enzyme activity to any extent (*data not shown*). Accordingly, the effects of the alternative diphosphoryl group donors, or nucleoside 5′-diphosphate or monophosphate inhibitors, were solely due to changes in *Mt*PRS enzyme activity.

### ITF Spectroscopy

**Figure 7:**
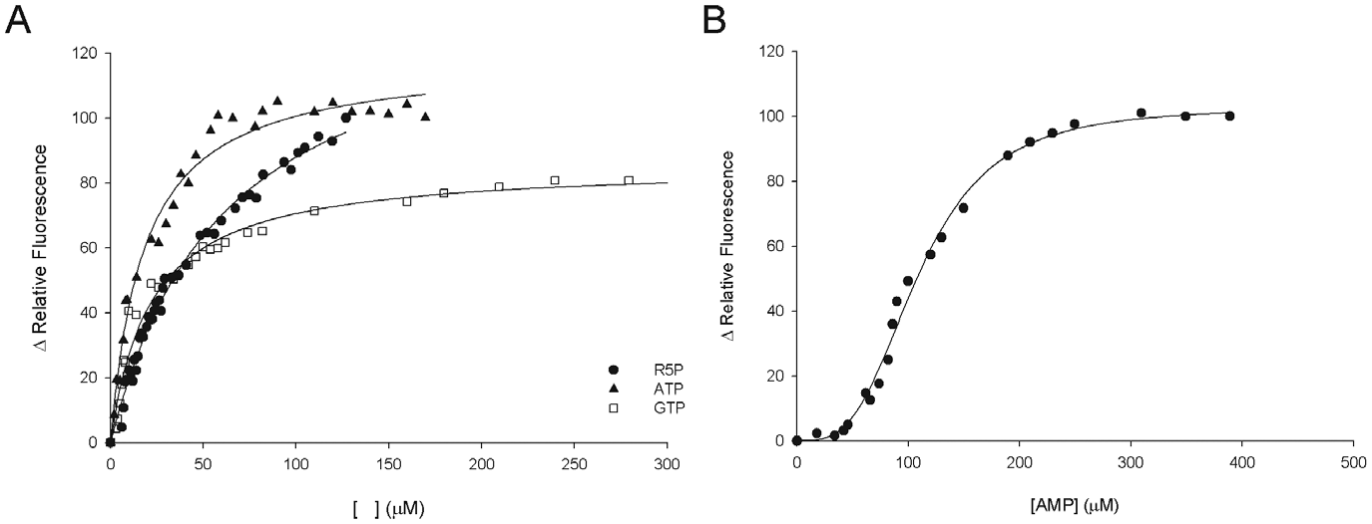
A) Hyperbolic equilibrium binding of R5P, ATP and GTP to MtPRS assessed by ITF. B) Sigmoidal equilibrium binding of AMP to *Mt*PRS assessed by ITF.

Binary complex formation between substrates (R5P, ATP, GTP) or products (AMP, PRPP) and *Mt*PRS was assessed by equilibrium fluorescence spectroscopy to ascertain the order (if any) of addition of these chemical compounds. Titration of *Mt*PRS with R5P, ATP and GTP were hyperbolic (**Figure 7A**). These data were thus fitted to **Eq. (5)**, yielding *K*_D_ values of 61 (±3) µM for R5P, 18 (±2) µM for ATP, and 21 (±2) µM for GTP. Titration of *Mt*PRS with AMP product was sigmoidal (**Figure 7B**), and data fitting to **Eq. (6)** yielded a value of 109 (±3) µM for *K’*. There was no intrinsic protein fluorescence change upon PRPP binding to *Mt*PRS, suggesting that PRPP cannot bind to free enzyme. Binding experiments were also carried out in an attempt to determine whether or not there is binary complex formation between *Mt*PRS and the alternative pyrimidine substrates UTP and CTP, which can also substitute for ATP as diphosphoryl group donors. No change in protein fluorescence could be detected upon titration of *Mt*PRS enzyme with UTP and CTP.

## Discussion

Recently, Alderwick and co-workers [30] and Lucarelli and co-workers [31] have also reported biochemical characterization of *Mt*PRS. Both reported protocols for cloning and purification of recombinant *Mt*PRS are significantly different from the one described herein, since *Mt*PRS reported here was produced as a non-His-tagged protein. Although many protocols use histidine tags to facilitate protein purification by the nickel-affinity chromatography strategy, adding histidine tags may alter the protein structure and the biological activity [47], [48]. We have thus deemed appropriate to make efforts to produce recombinant *Mt*PRS without any fusion partner to avoid any possible effect that the latter may have on the former. Notwithstanding, it should be pointed out that steady-state kinetics results were shown by Lucarelli and co-workers [31] to be quite similar for His-tagged *Mt*PRS as compared to *Mt*PRS treated with protease for removal of the N-terminal His-tag fusion partner. The three-step chromatographic purification protocol of recombinant *Mt*PRS here described yielded 3.5 mg of homogenous protein from 4 g of wet cell paste (**Figure 2A** and **Table 1**). Recombinant *Mt*PRS protein was stable at −80°C in the absence of additives. However, homogeneous *Mt*PRS could not be concentrated above 1 mg mL^−1^ in Tris HCl 50 mM pH 7.5 without precipitation, and activity of precipitated protein could not be recovered. Interestingly, Alderwick and co-workers [30] showed that recombinant C-terminal His-tagged *Mt*PRS was stable in solution up to 2 mg mL^−1^ in KH_2_PO_4_ buffer at pH 7.9 containing 150 mM NaCl, 1 mM DTT, 10% glycerol. It has been reported that addition of ammonium sulfate or Mg^2+^•ATP was needed to preserve 20% of *Mt*PRS activity in 50 mM Tris-HCl pH 8.0 and 50 mM Hepes-NaOH pH 8.0 buffers [31]. Lucarelli et al. [31] also reported that full activity of *Mt*PRS could be maintained with addition of 50 mM P_i_. No loss of activity could be observed for *Mt*PRS in Tris HCl 50 mM pH 7.5 buffer for the protein preparation here described. The possible explanations for these conflicting experimental observations are rather elusive at the moment.

*Mt*PRS quaternary structure could not be unequivocally determined by size exclusion liquid chromatography, in agreement with previous reports on PRS enzymes showing a tendency of these proteins to exist in multiple aggregated states in solution, ranging from dimeric to octameric quaternary structures [49], [50]. Accordingly, the glutaraldehyde cross-linking method followed by SDS-PAGE analysis [35] method was employed to determine the *Mt*PRS protein oligomerization state in solution. These data suggest that recombinant *Mt*PRS may adopt multiple oligomeric states over time, in which the homo hexameric form is the predominant quaternary structure for apo *Mt*PRS after 45 min incubation time in absence of P_i_. Addition of 50 mM P_i_ to apo *Mt*PRS seems to delay this quaternary structure organization shift from monomer to hexamer (**Figure 3A**). This apparent delay might be related to P_i_ mediated stabilization of recombinant *Mt*PRS at alternative organization of quaternary states (in dimeric or trimeric structures), as inorganic phosphate concentration of at least 25 mM has been proposed as essential for PRS complete stability [28]. Alternatively, binding of P_i_ to *Mt*PRS may lead to protection of lysine, tyrosine, histidine and arginine residues, slowing the reaction of glutaraldehyde cross-linking over time. However, whether the presence of P_i_ results in oligomeric state stabilization or in reduction in cross-linking remains to be established. Addition of R5P 5 mM appears to have no effect on time-dependent shifting of *Mt*PRS oligomeric states when compared to its apo form (**Figure 3B**). Nevertheless, *Mt*PRS incubation with ATP 5 mM (**Figure 3C**) leads to a shift towards dimeric quaternary structure with concomitant reduction in the hexameric form, whereas incubation with ADP 5 mM (**Figure 3D**) enhanced the *Mt*PRS tetrameric organization. These results are in agreement with previous reports on *M. tuberculosis* recombinant PRS quaternary structure (**Table 3**). Sedimentation velocity experiments in 50 mM KH_2_PO_4_ pH 7.9 buffer containing either R5P, ATP and ADP at the same concentrations described here led to somewhat similar changes in oligomerization states of *Mt*PRS [30]. Namely, R5P has no effect and ATP increased the hexameric species with concomitant decrease in homodimeric state of *Mt*PRS [30]. On the other hand, ADP has been reported to affect the molar mass distribution increasing the hexameric state with a concomitant reduction in trimeric species [30]. This shift could be related to human PRS isoform 1 [11] and *B. subtilis* [22] ADP binding site identification on the interface of three subunits in the hexamer, a quaternary structure that might be stabilized by the presence of ADP in solution. Interestingly, analytical gel filtration results suggested that apo *M. tuberculosis* PRS eluted as a single symmetrical peak consistent with the hexameric state in phosphate buffer [31]. The data here presented on glutaraldehyde cross-linking (**Figure 3A**) and elution of a single peak from Superdex 200 size exclusion column (protein purification protocol) suggest that *Mt*PRS exists as a hexamer in Tris HCl buffer and absence of ligands. Quaternary structure assignment of PRS enzymes in solution remains ambiguous with varying results in presence and absence of ligands [50]. It has been proposed that ADP binding to an allosteric site of *Mt*PRS induces stabilization of an inactive, hexameric oligomeric species [30]. Further efforts appear thus to be warranted to ascertain whether or not the dynamic equilibrium of *Mt*PRS has any bearing on enzyme activity.

**Table 3:** Comparison of biochemical data on *M. tuberculosis* PRS.

Reference	Activity assay	[P_i_] dependency	Cation dependency	Quaternary structure
**Alderwick, et al.** **2011** [30]	R5P + ATP → PRPP + AMP (PRS)AMP + ATP → 2ADP (MK)ADP + PEP → 2 piruvate +2 ATP (PK)2 piruvate +2 NADH +2H → 2 lactate +2 NAD^+^ (LDH)37°CKH_2_PO_4_ 50 mM pH 8.0	50 mM (full activation)5 mM (partial activity)	Activation: Mn^2+^ > Mg^2+^Inhibition: Ca^2+^	Analytical ultracentrifugation:E: trimer and hexamer (equilibrium);E + R5P 5 mM: trimer and hexamer;E + ATP 5 mM: ↓ hexamer ↑ dimer;E + ADP 5 mM: ↓trimer ↑ hexamer.
**Lucarelli, et al.** **2010** [31]	HPLC, AMP formation37°CKH_2_PO_4_ 50 mM pH 8.0	10–40 mM (59.7 U mg^−1^)	Activation: Mn^2+^ > Mg^2+^Inhibition: Cu^2+^ > Fe^2+^ > Ca^2+^	Size exclusion gel filtration:Hexamer (220 KDa).
**Breda, et al.** **2012** (this work)	R5P + ATP → PRPP + AMP (*Mt*PRS)PRPP + OA → PP_i_ + OMP (*Mt*OPRT)25°CTris HCl 50 mM pH 8.0	Activity without P_i_(1.16 U mg^−1^)	–	Cross-linking:E: monomer to hexamer;E + R5P 5 mM: monomer to hexamer;E + ATP 5 mM: ↓ hexamer ↑ dimer;E + ADP 5 mM: ↑ tetramer.

PRS enzyme activity is often assessed by radiochemical assays with either [^14^C]-R5P [30] or [γ-^32^P]-ATP detection [11], [25], [49], [51], by enzyme coupling with myokinase, pyruvate kinase and lactate dehydrogenase [52], or by a recently developed HPLC-based method that follows AMP formation [31]. Here we present, to the best of our knowledge, a novel coupled continuous spectrophotometric assay that measures the decrease in orotate concentration catalyzed by *Mt*OPRT in the presence of PRPP formed in solution by *Mt*PRS enzyme activity, a assay first proposed as a suitable alternative to follow PRS activity in the late 70′s, by Switzer and co-workers [28].

*Mt*PRS-catalyzed PRPP formation could be measured in the presence of R5P and ATP in absence of P_i_ (**Figure 4A**). Interestingly, it has been reported that P_i_ is required for *Mt*PRS enzyme activity [30], [31]. The reason for this discrepancy is not apparent at the moment. Measurements of *Mt*PRS enzyme activity here presented were carried out in the complete absence of P_i_ since the enzyme was stored in Tris HCl 50 mM pH 7.5 and activity measurements assessed in Tris HCl 50 mM MgCl_2_ 20 mM pH 8.0, OA 300 µM, *Mt*OPRT 1.8 U, and varied concentrations of ATP and R5P. It is possible that an explanation for this discrepancy may be attributed to coupled assay sensitivity, allowing *Mt*PRS activity detection even in absence of P_i_. In addition, the steady-state kinetic constants for *Mt*PRS enzyme reported in this work (**Table 2**) are considerably distinct from previous reports (**Table 4**) [30], [31]. It could be argued that the rather low values for the kinetic constants reported here are not representative of *Mt*PRS full activity, as it has being described that lower P_i_ concentrations led to partial enzyme activity [30]. However, it appears more plausible that the rather low value for the *Mt*PRS catalytic constant is due to the limiting value for the maximum velocity of the coupled enzyme (0.6 s^−1^) as reported elsewhere [38]. Notwithstanding, the results presented here demonstrate that P_i_ is not an obligatory requirement for *Mt*PRS catalytic activity. An interesting feature was identification of substrate inhibition by R5P when it is varied in the presence of saturating ATP concentration (**Figure 4B**), with K_i_ value of 211 µM. Substrate inhibition by R5P has been reported for rat liver PRS [53], as well as for *E. coli* [54] and *M. tuberculosis* [30], both in the presence of non-saturating P_i_ concentrations.

**Table 4:** Comparative of apparent kinetic parameters for *Mt*PRS substrate pair ATP/R5P.

Reference	Kinetic parameters				
	*K_M_* (µM)	*V_max_* (µmol^−1^ min^−1^ mg^−1^)	*k_cat_* (s^−1^)	*k_cat_/K_M_* (M^−1^ s^−1^)	*K_i_* (µM)
	**R5P**				
**Alderwick, et al. 2011** [30]	8.2	530	60.68	7430×10^3^	–
**Lucarelli, et al. 2010** [31]	71	–	37.1	521×10^3^	–
**Breda, et al. 2012** (this work)	14 (±2)	1.41 (±0.07)	0.83 (±0.04)	59 (±8)×10^3^	211 (±28)
	**ATP**				
**Alderwick, et al. 2011** [30]	S_0.5_ = 62.65/n = 1.68	601	–	–	–
**Lucarelli, et al. 2010** [31]	S_0.5_ = 260 (±50)/n = 1	–	34.6 (±3)	133.1×10^3^	–
**Breda, et al. 2012** (this work)	25 (±4)	1.12 (±0.03)	0.66 (±0.02)	26 (±4)×10^3^	–

**Figure 8:**
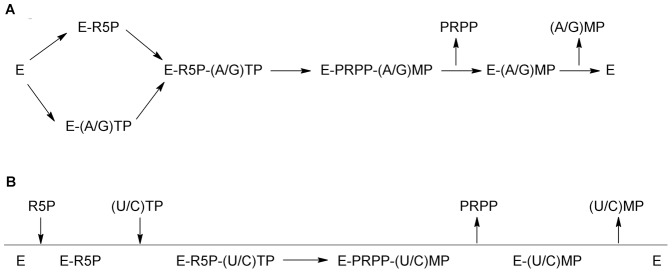
Proposed kinetic mechanisms for *Mt*PRS using A) purine or B) pyrimidine 5′-trisphosphate nucleotides, as diphosphoryl group donors. Random order of addition for ATP or GTP and ordered addition of UTP or CTP substrates, with ordered release of products.

The dependence of *Mt*PRS activity upon varying Mg^2+^ concentrations could not be assessed as this cation is essential for *Mt*OPRT coupled enzyme activity [38]. We have thus fixed the Mg^2+^ concentration at 20 mM based on both the optimum concentration for activity of *Mt*OPRT (larger concentration values are inhibitory) [38] and previously reported saturation curve for the dependence of *Mt*PRS activity on increasing Mg^2+^ concentration [31]. It has been shown that the enzyme requires free Mg^2+^ as an activator and as Mg^2+^•ATP co-substrate, and that free Mg^2+^ is likely to be an allosteric effector of the K-type enzyme model for cooperativity [31].

Substrate specificity measurements showed that *Mt*PRS can accept GTP, CTP, and UTP, in addition to ATP, as diphosphoryl group donors (**Figure 5**, **Table 2**), thereby showing broad substrate specificity. Although the *K_M_* values are similar, the values for the catalytic rate constants of GTP, CTP and UTP are lower than ATP, *Mt*PRS main substrate. *S. typhimurium* PRS, another bacterial PRS, has been described as being specific for ATP, although capable of using GTP, ITP, CTP and UTP as alternative substrates to a lesser extent (3% of maximum reported activity using ATP as substrate) [28].

The purine nucleoside diphosphates ADP and GDP were reported as PRS allosteric inhibitors [23], [25]. Under assay conditions here described, both behave partial inhibitors with IC_50_ values of, respectively, 0.07±0.01 mM (**Figure 6A**) and 0.9±0.1 mM (**Figure 6B**). ADP has been shown to be a non-competitive inhibitor of *Mt*PRS with overall inhibition constant values ranging from 320 µM to 522 µM [30]. On the other hand, it has been reported an IC_50_ value ranging from 0.26 mM (at saturating ATP and non-saturating P_i_ concentrations) to 0.4 mM (saturating ATP and saturating P_i_ concentrations) for ADP and an IC_50_ larger than 5 mM for GDP inhibition of *Mt*PRS activity in the presence of P_i_ [31]. These results prompted the proposal of a regulatory site to which both ADP inhibitor and P_i_ activator can bind [31]. Accordingly, ADP binding to the regulatory site hinders P_i_ binding resulting in inhibition of *Mt*PRS enzyme activity [31]. In addition, the sigmoidal curve for ADP inhibition of *Mt*PRS has been shown to affect the maximum velocity only, without affecting the value of *K*′ and the degree of cooperativity [31]. To evaluate if *Mt*PRS activity was responsive to pyrimidine regulation, the effect of UMP titration upon standard activity assay (Methods) was assessed (**Figure 6C**), which yielded an IC_50_ value of 3.0 (±0.8) mM. These findings seem to indicate that *Mt*PRS activity is regulated in response to *M. tuberculosis* energetic demand, being more responsive to purine inhibition (ADP and GDP), although variation in pyrimidine intermediates (UMP) could also regulate its activity. The regulation of *Mt*PRS enzyme activity by variations in both purine and pyrimidine intermediates is in accordance with the role of PRPP as a key intermediate in the metabolic pathways for the synthesis and recycling of both purine and pyrimidine nucleotides.

Hyperbolic binding isotherms determined from ITF measurements indicated that substrates R5P (*K*_D_  = 61 µM), ATP (*K*_D_  = 18 µM), and GTP (*K*_D_  = 21 µM) can bind to free apo *Mt*PRS (**Figure 7A**). Dissociation constant values for ATP and GTP are somewhat similar, an indicative that there might be no substrate preference between these purine nucleosides 5′-triphosphates, which is in agreement with their similar *K_M_* values (**Table 2**). Although the results of steady-state kinetic experiments have shown that CTP and UTP can act as diphosphoryl group donors, no change in IFT could be detected in the absence of R5P substrate. These findings might suggest that binding of pyrimidine nucleosides 5′-triphosphate results in no change in tryptophan fluorescence or that there is an alternative order of substrate addition for pyrimidine nucleotides. No change in ITF could be detected upon PRPP titration into free *Mt*PRS. On the other hand, AMP product showed hyperbolic variation of ITF upon titration to free *Mt*PRS, with *K*′ value of 109 µM and a Hill coefficient value of 3.2 (**Figure 7B**), an indicative of positive homotropic cooperativity. Further experiments of isothermal titration calorimetry could be pursued to address the issue of whether or not pyrimidine nucleotides are capable of binding to free *Mt*PRS.

Data on steady-state kinetics and equilibrium binary complex formation suggest that the enzyme mechanism of *Mt*PRS for purine (ATP and GTP) diphosphoryl donors follows a random-ordered substrate addition with ordered product dissociation, in which PRPP is the first product to be released followed by purine nucleoside monophosphate products (AMP or GMP) to yield free enzyme for the next round of catalysis (**Figure 8A**). Although the order of substrate addition can be proposed as it is based on solid experimental evidence, the order of product release has to be considered with caution. For instance, it is possible that PRPP binding to free *Mt*PRS enzyme results in no change in ITF, which would imply in a random-order mechanism of product release. Isothermal titration calorimetry can thus be used to address the issue of whether or not PRPP is capable of binding to free *Mt*PRS. The enzyme mechanism for pyrimidine (UTP or CTP) diphosphate donors might obey an ordered mechanism of substrate addition and product release; in which R5P binds to free enzyme followed by the diphosphate donors, and PRPP release is followed by pyrimidine nucleoside monophosphate products (UMP or CMP) to yield free *Mt*PRS (**Figure 8B**).

Considering their molecular and kinetic characterization, three different classes of PRS enzymes have been described. Classifications of PRS proteins as belonging to Class I (also known as “Classical="). Class II or Class III are based on specificity for diphosphoryl donors, requirement of P_i_ for activity, allosteric inhibition by purine ribonucleoside diphosphates, and oligomeric states [23], [25], [31]. It has been proposed that there is also a proportional relationship among *K*_M_, *V*_max_ and PRS classes [25], in which Class III enzymes have larger *K*_M_ values for R5P and ATP substrates, Class I with the lowest values, and Class II with intermediate values [25]. The extent to which these criteria could be used for classifying PRS enzymes are still not clear due to limited number of representatives of Classes II and III PRSs [25].

*Mt*PRS has approximately 41% identity to the three human PRS isoforms, as well as to *A. thaliana* and spinach Class I enzymes (isoforms 1 and 2). The degree of primary sequence conservation drops to 18–23% when the *M. tuberculosis* sequence is compared to Class II PRS enzymes from the latter two organisms (isoforms 3 and 4). As previously demonstrated [11], [25], [31], the amino acids involved in substrate binding are the most conserved regions: *Mt*PRS Tyr88-Ser104 and Asp166-Arg169 for ATP binding, and *Mt*PRS Asp219-Thr227 for R5P binding. All amino acids involved in ADP allosteric site, according to *B. subtilis* quaternary structure [21], are conserved in *Mt*PRS (Ser43, Arg45, Ser77, Ala78, Lys96, His97, Arg98, Gly99, Arg100, Gln131, Asp139, His140, Ser306 and Phe311), in agreement with the inhibition data presented in **Figure 6A** and with previous reports showing that ADP is an allosteric inhibitor of *Mt*PRS [30], [31]. Despite low amino acid conservation, secondary structure prediction showed that homotrimeric spinach PRS isozyme 4 (a Class II enzyme) and hexameric *B. subtilis* PRS (a Class I enzyme) have a similar folding pattern [23]. No Class II PRS structure has been solved so far, thus any inferences about amino acids substitution that might account for this class broader substrate specificity are, based on available structural data, somewhat speculative. PRS nucleotide binding pocket is located in a wide cleft, and the secondary structure elements might undergo conformational rearrangements upon ligand binding to accommodate both purine and pyrimidine bases, as well as properly positioning of amino acids side chains to specifically hydrogen bond each diphosphoryl group donor.

The broad specificity for diphosphoryl group donors and detection of enzyme activity in the absence of P_i_ would suggest that *Mt*PRS belongs to Class II PRS proteins. On the other hand, the hexameric quaternary structure assembly, as suggested by cross-linking experiments (**Figure 3**) would indicate that it belongs to Class I PRS enzymes. In addition, allosteric inhibition by ADP [30], [31] (**Figure 6A**) would place *Mt*PRS in Class I PRSs. Accordingly, it has previously been suggested that *Mt*PRS belongs to Class I [30]. Further data are thus needed to classify *Mt*PRS as belonging to a particular family of PRS proteins.

It should be pointed out that the results here presented extend previous studies on *Mt*PRS [30], [31]. To the best of our knowledge, the results here presented are, along with *S. typhimurium* PRS data, the first experimental evidence for a bacterial PRS enzyme that can use both pyrimidine and purine nucleosides triphosphate as diphosphoryl group donors since broad substrate specificity has been described for plants only. In addition, this is the first report on *Mt*PRS enzyme mechanism for purine and pyrimidine diphosphate donors. Current efforts are towards experimental structure determination of *Mt*PRS to provide a solid foundation for the rational design of, hopefully, specific inhibitors of this enzyme without affecting to a great extent the host PRS.
